# Reference des cas de traumatisme par arme à feu en Afrique

**DOI:** 10.5588/pha.23.0006

**Published:** 2023-08-01

**Authors:** M. G. Tito, J. P. K. Makelele, W. van den Boogaard, S. Ade, A. Deselets, E. Briskin, C. Badjo, D. Salviati, E. T. Akem, M. Hejdenberg

**Affiliations:** 1 Médecins Sans Frontières (MSF) Centre Opérationnel Amsterdam, Amsterdam, Pays-Bas; 2 MSF – Centre Opérationnel Bruxelles, Kinshasa, DR Congo; 3 MSF-Luxembourg, centre Opérationnelle Bruxelles, Département Médicale, Unité recherche opérationnelle (LuxOR), Ville de Luxembourg, Luxembourg; 4 Faculté de Médecine, Université de Parakou, Parakou, Bénin; 5 University for Peace (UN-mandated) - Department of International Law, Costa Rica; 6 MSF – Epicentre, Paris, France

**Keywords:** conflit armé, pays en faible revenue, référence, recherche opérationnelle, violence

## Abstract

**CONTEXTE:**

Dans un pays d’Afrique émaillé de violences de guerre, Médecins Sans Frontières appui deux hôpitaux régionaux de références, pour répondre aux urgences incluant le traitement des traumatismes par arme-à-feu. Il facilite l’accès aux soins et références qui sont régulièrement entravés pour des raisons non médicales.

**OBJECTIF:**

Déterminer les facteurs influençant l’issue défavorable des cas référés pour traumatisme par arme-à-feu (décembre 2020–novembre 2021).

**TYPE D’ÉTUDE:**

Ceci est une étude transversale utilisant des données collectées de routine.

**RÉSULTATS:**

Au total, 381 patients, victimes de traumatisme par arme-à-feu étaient admis avec une moyenne d’âge de 29 ans ; 28,3% étaient des lésions des gravités sévères dont les lésions thoraco-abdominale et les fractures. La mortalité était 4,9% et les sortis contre-avis médical 7,9%. Les patients d’affiliation force irrégulière représentaient 45,4%, et avaient deux sur trois fois une référence non-aboutie pour des raisons non-médicales. Les patients avec des lésions sévères au triage : l’affiliation, force irrégulière et armée régulière avaient respectivement 2 (*P* < 0,01), 5,9 (*P* < 0,01) et 8,1 (*P* < 0,01) fois plus de risque d’avoir une issue défavorable.

**CONCLUSION:**

Les cas graves causés par des blessures par arme-à-feu risquaient d’avoir plus d’une issue défavorable. Ceci était amplifié pour ceux d’une certaine affiliation, qui se voyaient plus souvent refuser l’accès aux soins de référence supérieur plutôt basé sur des raisons sociopolitiques que sur des raisons médicales.

## Abstract (en)

**BACKGROUND:**

In a war-torn African country, Médecins Sans Frontières supports two regional referral hospitals to address emergencies, including the treatment of firearm-related traumas. It facilitates access to healthcare and referrals, which are often hindered due to non-medical reasons.

**OBJECTIVE:**

To determine the factors influencing the unfavourable outcome of cases referred for firearm trauma (December 2020–November 2021).

**METHOD:**

This was a cross-sectional study using routinely collected data.

**RESULTS:**

A total of 381 patients who were victims of firearm-related trauma were admitted, with an average age of 29 years; 28.3% had severe injuries, including thoraco-abdominal injuries and fractures. The mortality rate was 4.9%, and 7.9% left against medical advice. Patients affiliated with the irregular armed forces accounted for 45.4%, and had a two-thirds higher rate of unsuccessful referral for non-medical reasons. Patients with severe injuries at triage, affiliation with the irregular forces and being in the regular army had 2 times (*P* < 0.01), 5.9 times (*P* < 0.01) and 8.1 times (*P* < 0.01), respectively, a higher risk of an unfavourable outcome.

**CONCLUSION:**

Serious cases caused by firearm injuries were more likely to have an adverse outcome. The risk was higher in those with a specific affiliation, who were more often denied access to higher referral care based on socio-political rather than medical reasons.

Les traumatismes par arme à feu (TAF) constituent un véritable problème de santé publique dans le contexte de la violence et de l’insécurité dans les pays à revenu faible ou intermédiaire (PRFI).^1^ Ils sont à l’origine d’un nombre élevé d’admissions dans les services d’urgence des établissements de santé et d’une augmentation du taux de mortalité.^2^

Les éléments déterminants pour une prise en charge adéquate des TAF sont multiples, incluant entre autres un diagnostic précoce, une évaluation correcte de la gravité et la disponibilité de ressources nécessaires à une stabilisation du patient à son admission.^3–9^ A l’opposé, les recours aux soins et les références tardives ainsi que l’insuffisance du plateau technique influencent négativement le pronostic des TAF.^3,4,6^

A cela, pourraient parfois s’ajouter en situation conflictuelle, la violation du droit international humanitaire (DIH) qui garantit l’accès aux soins de santé sans discrimination et celle du droit de protection spécifique adapté à la situation de violence.^10,11^ Une telle situation entrainerait une fréquence plus élevée d’issues défavorables chez les victimes incluant entre autres, une plus forte mortalité, ou encore des références non abouties. Cependant, malgré la richesse de la littérature existante, on dispose peu d’informations sur l’impact des facteurs non médicaux sur le pronostic des TAF dans une situation de conflit armé. En plus, les littératures disponibles sont basées sur des études essentiellement menées dans les pays à revenus élevés ou intermédiaires.^12,13^ Peu de données en provenance des pays à faibles revenus (PFR) sont disponibles, alors que nombreux d’entre eux ne sont pas épargnés par l’insécurité.^12^

Médecins Sans Frontières (MSF) appui les services de chirurgie des deux hôpitaux régionaux et les références au niveau tertiaire dans un de ce pays d’Afrique subsaharienne (tenue anonyme pour éviter l’exposition du personnel soignants et les personnes impliquées dans l’étude) à faibles revenus, en proie depuis plus d’une décennie à une instabilité politique et des conflit armés,

Cette étude vise à décrire et déterminer chez les patients référées et admis pour TAF entre décembre 2020 et novembre 2021: 1) leurs caractéristiques sociodémographiques et cliniques ; 2) les conditions de déroulement de la référence et son résultat ; 3) les issues d’hospitalisation des cas référés ; et 4) identifier les facteurs associés d’une part à une référence non aboutie, et d’autre part à une issue défavorable.

## METHODE D’ETUDE

### Cadre

Dans ce pays, les combats armés sont récurrents dans différentes régions. MSF appui le Ministère de la Santé dans deux hôpitaux régionaux pour faciliter l’accès aux soins et la prise en charge. Ces hôpitaux sont régulièrement confrontés à une augmentation des admissions pour TAF lors des épisodes des violences liées à la guerre, braquage, règlement des comptes et homicides involontaires entre autres

Les patients qui sont admis dans les hôpitaux régionaux (niveau de référence II) proviennent des hôpitaux des districts, centres de santé et périphérique (niveau de référence I) suivant ou non le système de référence, utilisant les véhicules, moto, vélo ou même les pieds comme moyen de transport.

Ils bénéficient au niveau II, des soins immédiats de stabilisation et de prise en charge spécifique selon les critères du « Système d’échelle de triage sud-africain »,^14^ qui donne un score de sévérité en quatre couleurs, dont la couleur verte regroupe les patients avec des lésions bénignes, mineures non graves ; la couleur jaune définie les patients graves à stabiliser avec nécessité d’un traitement supplémentaire ; la couleur rouge classe les patients avec des lésions très graves nécessitant des soins immédiats pour sauver leur vie ; alors que le noir classe les patients mourant ou arrivés morts.

Après les soins de stabilisation, certains patients sont référés au niveau national (niveau III) à la capitale pour des soins spécialisés par limite du plateau technique des niveaux II (voir Tableau 1 pour les détails des plateaux techniques des hôpitaux niveau II et III).

**TABLEAU 1:** Définition contextuelle du plateau technique des structures sanitaires de référence

Plateau technique sur les niveaux des hôpitaux régionale (niveau II) :
Dans les hôpitaux régionaux, la prise en charge des traumatismes par arme à feu est souvent limité par les limites du plateau technique chirurgical qui se définie à travers les éléments suivants :
Bloc opératoire : chaque hôpital contient un bloc opératoire mais l’un de deux à un état de fonctionnement très faible par manque personnel qualifiés et de la qualité de service de la prévention et control des infections
Equipement : les deux blocs ne sont pas équipés de manière adéquate : de moniteur, de C-arme pour une bonne traumatologie, la basse qualité et/ou l’absence des clichés radiographiques et matériels pour les ex-fix (matériel de fixateur externe adéquates)
Ressources Humaines : Absence des personnels qualifiés pour la chirurgie dans un des hôpitaux dont le service de chirurgie n’est pas appuyé par Médecins Sans Frontières (chirurgien et anesthésiste cimma etc.)
Plateau technique sur les niveaux des hôpitaux universitaire/nationaux (niveau III), dont
Médecins Sans Frontières réfère les patients :
Les hôpitaux universitaires de la capitale sont dotés des blocs opératoires, équipements et service de kinésithérapie appropriés pour la prise en charge traumato-orthopédique des patients avec un traumatisme par arme à feu de tout âge. Les personnels spécialistes qualifiés en chirurgies traumato-orthopédiques pédiatriques et adulte

A ce stade, selon les observations, le système de référence est confronté à un problème de sélectivité car les services de renseignements aéroportuaires font leur propre triage des patients parfois se basant sur l’affiliations socio-politique de la personne et non sur les besoins médicaux. Les affiliations sont regroupées en trois catégories : 1) civil pour les personnes non militaires qui ne sont pas activement concerné par les conflits, 2) armée régulière pour les forces armées gouvernementales, et 3) force irrégulière pour les groupes armés de résistance opposé au régime politique en place.

Ce phénomène limiterait par défaut, l’accès aux soins spécialisés pour certains patients avec comme conséquence que ceux qui sont affiliés à une « force irrégulière » quittent l’hôpital contre avis médical car ils craignent d’être capturés, ou poursuivis et de subir des représailles, ce qui est également décrit dans l’étude systématique de Haar et al.^15^ Les patients affiliés à l’armée régulière quittent également l’hôpital contre l’avis du médecin, mais parce qu’ils pensent recevoir des soins continus dans leur caserne après stabilisation, ce qui n’est pas nécessairement l’idéale. Nous considérons donc que les patients qui sont sortis d’eux-mêmes contre avis médical, ainsi que les patients qui sont décédés, ont eu une évolution défavorable.

### Type d’étude

Il s’agit d’une étude transversale avec collecte des données rétrospectives recueillies entre décembre 2020 et novembre 2021.

### Population

Tous les patients victimes de TAF admis dans les deux hôpitaux régionaux appuyés par MSF pendant la période étudiée étaient inclus.

### Variables et sources des données

Des informations sur les caractéristiques sociodémographiques et cliniques comme : affiliation sociopolitique, le type de violence, les lésions observées, le degré de gravité, la référence, ces modalités et le délai du temps, en suit le résultat et le devenir du patient ont été colligées. Elles ont été extraites de la base de données MS Excel (Microsoft, Redmond, WA, États Unis) renseignée en routine, qui incluait la consultation des dossiers des patients, puis anonymisées.

### Analyse statistique

Les données ont été analysées avec le logiciel Epi Info v7.2.4.0 (Centers for Disease Control and Prevention, Atlanta, GA, États Unis) et R v4.2.2 (R Computing, Vienne, Autriche). Les variables continues ont été décrites en utilisant la moyenne (±déviation standard) ; et les variables catégorielles en utilisant des pourcentages et des proportions. Les facteurs associés aux références non-abouti et à issue défavorable étaient étudiés par analyse bivariée, en déterminant l’odds ratio brut (ORb), son intervalle de confiance à 95% (IC 95%) et la valeur *P* < 0,05. Les facteurs avec une association significative dans l’analyse bivariée ont été inclus dans un modèle multivarié pour calculer les OR ajustes (ORa).

### Considérations éthiques

Cette recherche remplissait les critères d’exemption fixés par le Comité d’éthique de MSF (MSF Ethics Review Board), Génève, Suisse, pour les analyses a posteriori des données cliniques collectées de manière routinière et ne nécessitait donc pas d’examen par MSF-ERB. Elle a été menée avec l’autorisation du Directeur Médical du MSF Centre Opérationnel à Amsterdam, Pays Bas.

## RÉSULTATS

Au total, 381 patients victimes de TAF ont été admis dans les deux hôpitaux régionaux appuyés par MSF. Parmi eux, 342 (89,8%) étaient de sexe masculin, et l’âge moyen était de 29 ans. Trois-quarts de ces cas était au cause de la guerre (*n* = 279 ; 73,2%). Parmi eux, 189 (49,6%) étaient des civils, et 173 (45,4%) étaient des forces irrégulières (Tableau 2). Sur le plan clinique (Tableau 3), 108 (28,3%) patients étaient classés dans la catégorie rouge au triage des urgences de la référence du niveau I vers II dont 43 (48,3%) étaient référés du niveau II vers III. La moto était le mode de transport le plus fréquent 273 (71,7%) pour une référence du niveau I vers le niveau II. La lésion de partie molle était dans presque la moitié de cas (*n* = 173 ; 45,4%) diagnostiqués. Trois-quarts des cas avec une fracture (*n* = 67 ; 75,3%) était référés vers niveau III. Le délai médian des références du niveau I vers II était de 2 jours (intervalle interquartile 0–4). Pour 37 (41,6%) de cas qui ont eu besoin d’une référence vers le niveau III, celle-ci n’a pas eu lieu. Quarante-neuf (12,8%) des patients ont eu un devenir défavorable, dont 19 (4,9%) décès et 30 (7,9%) sortis contre avis médical au niveau II.

**TABLEAU 2:** Caractéristiques sociodémographiques des patients admis pour traumatisme par arme à feu référés vers les services de chirurgie des deux hôpitaux régionaux entre décembre 2020 et novembre 2021

	Effectif (*n* = 381) *n* (%)
Sexe	
Masculin	342 (89.8)
Féminin	39 (10.2)
Affiliation du patient	
Civile	189 (49.6)
Armée régulière	19 (5.0)
Force irrégulière	173 (45.4)
Type de violence	
Guerre	279 (73.2)
Braquage	44 (11.6)
Autre*	58 (15.2)
Age moyen ± DS	29 ± 11.6

*Règlement de compte, homicide involontaire, arme de chasse, etc.

DS = déviation standard.

**TABLEAU 3:** Caractéristiques médicales des patients admis pour traumatisme par arme à feu et référés du niveau I vers II (deux hôpitaux régionaux appuyés par MSF) et du niveau II vers III (quatre hôpitaux capital) entre décembre 2020 et novembre 2021

	Référence Niveau I vers II (*n* = 381) *n* (%)	Référence Niveau II vers III (*n* = 89) *n* (%)
Sévérité de triage		
Verte	39 (10.2)	—
Jaune	230 (60.4)	NA*
Rouge	108 (28.3)	NA*
Noire	4 (1.1)	—
Modalité d’arrivée		
Pieds	—	
Vélo	1 (0.3)	
Moto	273 (71.7)	
Véhicule	101 (26.5)	
Avion	—	89 (100)
Type de lésion		
Lésion de partie molle	173 (45.4)	8 (8.9)
Fracture	134 (35.2)	67 (75.3)
Lésion thoraco-abdominale	74 (19.4)	14 (15.7)
Délais de référence, jours, médiane [IQR]	2 [0–4]	DM*
Référence aboutie		
Non	NA^†^	37 (41.6)
Oui	NA^†^	52 (58.4)
Issue après traitement		
Guéri	276 (72.4)	DM*
Sortie contre avis médical	30 (7.9)	DM*
Décédé	19 (4.9)	DM*
Référé	51 (13.4)	NA^‡^

MSF = Médecins Sans Frontières ; DM = données manquantes ; NA = non applicable ; IQR = *interquartile range* (intervalle interquartile).

*Parce que le système de triage est à l’admission du niveau II.

^†^Parce qu’au niveau II, seuls les patients qui sont venus sont inclus, il n’y a donc pas de non-abouti.

^‡^Parce qu’il n’y a plus d’autre autre niveau pour référer des patients.

Les facteurs associés à un risque élevé d’issue défavorable ­(Tableau 4A) étaient d’affiliation non civil du patient, dont l’armée régulière (ORa 10.6 ; IC 95% 2,7–40.9 ; *P *≤ 0,01), puis la force irrégulière (ORa 8.3 ; IC 95% 3.6–22.6 ; *P* < 0,01), et la gravité des lésions, notamment de catégorie rouge (ORa 2.0 ; IC 95% 1,0–3,9 ; *P* < 0,01).

**TABLEAU 4 A):** Facteurs associés à l’issue défavorable chez les patients (*n* = 381) admis pour traumatisme par arme à feu référés du niveau I vers II entre décembre 2020 et novembre 2021

	Analyse bivariée				Analyse multivariée
	Issue défavorable (*n* = 49) *n* (%)	Issue favorable (*n* = 332) *n* (%)	*P*-value	ORb (IC 95%)	ORa (IC 95%)
Age moyen ± DS	29.7 ± 8.6	28.8 ± 11.9	0.5	1.0 (1.0–1.0)	—
Sexe			0.2		
Féminin	2 (4.1)	37 (11.1)		Référence	—
Masculin	47 (95.9)	295 (88.9)		2.9 (0.7–26.0)	—
Délais de référence, h			0.3		
<24	12 (24.5)	112 (33.7)		Référence	—
≥24	37 (75.5)	220 (66.3)		1.6 (0.8–3.4)	—
Affiliation du patient			<0.01*		
Civile	8 (4.2)	181 (95.8)		Référence	
Armée régulière	5 (26.3)	14 (73.7)		8.1 (2.2–27.7)	10.6 (2.7–40.9)
Force irrégulière	36 (20.8)	137 (79.2)		5.9 (2.8–14.1)	8.3 (3.6–22.6)
Type de lésion					
Lésion de partie molle	20 (40.8)	153 (46.1)	0.22	Référence	—
Fracture	15 (30.6)	119 (35.8)		0.9 (0.4–1.9)	—
Lésion thoraco-abdominale	14 (28.6)	60 (18.1)		1.7 (0.8–3.7)	—
Sévérité de triage					
Verte	0 (0.0)	39 (11.7)	<0.01*	0	0
Jaune	25 (51.0)	205 (61.7)		Référence	Référence
Rouge	20 (40.1)	88 (26.5)		1.9 (1.0–3.5)	2.0 (1.0–3.9)
Noire	4 (8.1)	0 (0.0)		NA	NA

*Fisher’s Exact.

ORb = odd ratio brut ; IC = intervalle de confiance ; ORa = odd ratio ajusté ; DS = déviation standard ; NA = non applicable.

Sur les 89 patients référés du niveau II vers III (Tableau 4B), les patients d’affiliation « force irrégulière » étaient plus à risque d’une référence non aboutie que ceux ayant un statut civil ou « armée régulière » (*P* < 0,01). L’affiliation de force irrégulière avait une proportion de 100% d’une référence non-abouti vers niveau III tandis que 0% des personnes des autres affiliations n’avaient des références non abouties. Il n’y a pas une différence significative associée entre la gravité des lésions et la référence.

**TABLEAU 4 B):** Facteurs associés à la référence non aboutit chez les patients (*n* = 89) admis pour traumatisme par arme à feu référés du niveau II vers III entre décembre 2020 et novembre 2021

	Référence non aboutie (*n* = 37) *n* (%)	Référence aboutie (*n* = 52) *n* (%)	*P*-value Fisher Exact	ORb (IC 95%)
Age moyen ± DS	29.5 ± 7.1	29.9 ± 11.3	0.8	
Sexe			1	
Féminin	1 (2.7)	1 (1.9)		Référence
Masculin	36 (97.3)	51 (98.1)		0.71 (0.0–113.2)
Affiliation du patient			<0.01	
Civile	0 (0.0)	30 (57.7)		0 (ND)
Armée régulière	0 (0.0)	6 (11.5)		Référence
Force irrégulière	37 (100)	16 (30.1)		Infini
Type de lésion			0.3	
Partie molle	4 (10.8)	4 (7.7)		Référence
Fracture	30 (21.1)	37 (71.2)		0.83 (0.17–3.3)
Thoraco-abdominale	3 (8.1)	11 (21.2)		0.72 (0.04–1.67)

ORb = odd ratio brut ; IC = intervalle de confiance ; DS = déviation standard ; ND = non disponible.

## DISCUSSION

Cette étude est la première dans ce pays d’Afrique qui aborde les aspects qui influencent le devenir des patients dans un contexte si violent des récurrentes guerres. Notre population d’étude était majoritairement des jeunes hommes (âge moyenne : 29 ans) dont la moitié était d’une affiliation civile. Parmi l’affiliation « force irrégulière » seulement un sur trois qui avaient besoins d’une référence vers le troisième niveau avait aboutie. Le taux d’issue défavorable était de 12,8% dont 4,9% des décès et 7,9% de sortie contre-avis médical quelle que soit leur gravité, y compris les fractures qui étaient les plus courantes pour les besoins de référence vers le niveau III.

### Délai du temps en relation avec le mode de référence et la conséquence

La plupart des patients ont utilisé des motos et des véhicules de différentes origines sur des routes dans un état de délabrement avancé comme moyen d’arrivée à l’hôpital de référence de niveau II, ce qui est très similaire à d’autres milieux à faibles ressources.^16,17^ Ceci constitue un facteur d’accès tardif aux soins d’urgence et de référence, qui paradoxalement n’a pas influencé le devenir contrairement aux diverses autres publications qui le mentionnent comme un facteur déterminant.^7,8^

Au triage, plus d’un quart des patients à l’admission, avaient des lésions de sévérités grave (catégorie rouge). Prêt d’un patient sur trois avait une fracture dont la moitié des lésions des fractures ont plus bénéficié des références de niveaux tertiaires face à un peu moins d’un sur quatre avait eu des lésions thoraco-abdominales pour lesquelles quatre sur cinq n’ont pas bénéficiés de cette référence, probablement à cause de leur complexité dans la stabilisation et la prise en charge initiale.^8^

Les patients avec des lésions de sévérités graves avaient deux fois plus de risque d’avoir une issue défavorable, ce qui est conforme aux autres études.^4^ Par contre, le taux de mortalité était de 4,9%, qui semble très bas et est effectivement en opposition avec des autres études.^2^ Ce taux pourrait s’expliquer en parti, par des patients qui sont sorti contre avis médical (8%), qui ne sont pas pris en compte ni dans le survécu ni dans les décès, et parmi eux il y a très probablement un certain nombre qui serait décédé.

### Sélectivité à la qualité de soins lié à l’affiliation du patient

La sélectivité à la qualité des soins liée à l’affiliation du patient est illustrée de manière particulière : les patients de l’affiliation « armée régulière » et « force irrégulière » ont été confrontés à différents degrés d’accès et de qualité des soins selon qu’il s’agissait ou non de la continuité des soins après la sortie contre avis médical ou de l’accès aux soins au niveau national par les références qui sont l’influencés par la sélectivité faite par les services aéroportuaires.

Malgré que 30% des patients de l’affiliation force irrégulière ont eu une référence aboutie vers le troisième niveau, cette proportion reste très peu par rapport aux autres affiliations armée régulière et les civiles. Cette sélectivité des références des patients a entraîné des hésitations, voire des refus, avec une forte probabilité de non-adhésion au système de référence, parce que la confidentialité n’aurait pas été respectée. Ce phénomène est bien décrit par Haar et al.^15^ qui mentionnent malgré le peu de données disponibles, les références dans le contexte de la guerre, alors que d’autres littératures abordent sur des autres aspects de la violence : homicide volontaire ou involontaire, vol à main armée, etc.,^13^ qui ne montrent pas un impact pour ces patients sur le système de référence. Cette différence suggère une violation systématique du DIH pour les patients qui dans les conflits règlemente la sauvegarde de l’accès aux soins pour tous, y compris les individus qui ont pris part aux hostilités mais qui sont maintenant hors combat, sans aucune discrimination fondée sur des motifs autres que médicaux.^18,19^ La force de cette étude est le fait qu’elle prévaut être la première dans ce contexte à aborder l’issue des obstacles fondés sur l’accès limité aux soins en particulier d’un certain groupe des patients, qui étaient victime de la discrimination sélective au système de référence édicté par la volonté et considérations sociopolitiques de ce pays. Cette étude quantitative a pu démontrer la relation entre certaines variables, mais une future recherche qualitative dans ce contexte pourrait aider à documenter et mieux expliquer les raisons derrières ces tendances.

Cette étude a eu plusieurs limitations. Il y avait une difficulté d’accéder aux données des hôpitaux tertiaires, donc on n’a pas pu inclure toutes les issues réelles des patients à cause des données rétrospectives et les variables non disponibles dans la base de données malgré la recherche dans les dossiers des patients. En plus, la vraie issue des patients avec une sortie contre avis médical n’est pas connue, ce qui pourrait biaiser nos résultats. Onze dossiers des patients qui n’avaient presque aucune donnée sur les variables pertinentes étaient exclus de l’étude.

## CONCLUSION

Notre étude a mis en évidence plusieurs aspects liés aux patients souffrant de TAF et admis dans un pays en guerre. La gravité des lésions et le plateau technique des hôpitaux de niveau secondaire, les retards dans la présentation et l’accès aux soins du niveaux tertiaire d’une catégorie des patients posaient problème, surtout pour les « forces irrégulières », pour des raisons non médicales. Cela constitue une violation du droit international humanitaire, ainsi que de la loi constitutionnelle du pays. Dans un tel contexte, il est primordial pour les services médicaux de plaider pour un accès aux soins équitable a tous, sans discrimination autre que des besoins médicaux La mortalité était toutefois faible, et l’effet indésirable « sortie contre avis médical » était beaucoup plus élevé sans être pris en compte dans le devenir de ces patients.

### Remerciements

Nos remerciements vont à la mission du Centre Opérationnel Amsterdam du pays d’étude qui a facilité et rendu possible cette étude a différents niveaux ; centre opérationnel au comité scientifique et d’éthique d’Amsterdam, Pays Bas, et les différents adviseurs impliqués dans la validation du protocole en français pour cette étude ; et aux collègues de l’Initiative de recherche opérationnelle et de formation structurée (SORT-IT) pour les encouragements et motivations mutuelles, et à toute personne de près comme de loin impliqué dans la réalisation de l’étude.

Cette recherche a été menée dans le cadre SORT-IT, un partenariat mondial dirigé par le Programme spécial de recherche et de formation sur les maladies tropicales de l’Organisation mondiale de la santé (OMS/TDR). Le modèle est basé sur un cours élaboré conjointement par l’Union internationale contre la tuberculose et les maladies respiratoires (L’Union) et MSF. Le programme spécifique, SORT-IT, qui a donné lieu à cette publication a été organisé par MSF spécifiquement pour la recherche en langue française.

Le programme a été financé par La Fondation Veuve Emile Metz-Tesch, Luxembourg. Le financeur n’a joué aucun rôle dans la conception de l’étude, la collecte et l’analyse des données, la décision de publier ou la préparation du manuscrit.
